# Development of a Physiologically-Based Pharmacokinetic Model of the Rat Central Nervous System

**DOI:** 10.3390/pharmaceutics6010097

**Published:** 2014-03-18

**Authors:** Raj K. Singh Badhan, Marylore Chenel, Jeffrey I. Penny

**Affiliations:** 1 Manchester Pharmacy School, the University of Manchester, Oxford Road, Manchester, M13 9PT, UK; 2 EA 3809, UFR Médecine-Pharmacie, 34 Rue du Jardin des Plantes, BP 199, 86005 Poitiers, France

**Keywords:** physiologically-based pharmacokinetic model, blood–brain barrier, cerebrospinal fluid, unbound fraction, brain

## Abstract

Central nervous system (CNS) drug disposition is dictated by a drug’s physicochemical properties and its ability to permeate physiological barriers. The blood–brain barrier (BBB), blood-cerebrospinal fluid barrier and centrally located drug transporter proteins influence drug disposition within the central nervous system. Attainment of adequate brain-to-plasma and cerebrospinal fluid-to-plasma partitioning is important in determining the efficacy of centrally acting therapeutics. We have developed a physiologically-based pharmacokinetic model of the rat CNS which incorporates brain interstitial fluid (ISF), choroidal epithelial and total cerebrospinal fluid (CSF) compartments and accurately predicts CNS pharmacokinetics. The model yielded reasonable predictions of unbound brain-to-plasma partition ratio (*K*p_uu,brain_) and CSF:plasma ratio (CSF:Plasma_u_) using a series of *in vitro* permeability and unbound fraction parameters. When using *in vitro* permeability data obtained from L-mdr1a cells to estimate rat *in vivo* permeability, the model successfully predicted, to within 4-fold, *K*p_uu,brain_ and CSF:Plasma_u_ for 81.5% of compounds simulated. The model presented allows for simultaneous simulation and analysis of both brain biophase and CSF to accurately predict CNS pharmacokinetics from preclinical drug parameters routinely available during discovery and development pathways.

## 1. Introduction

Quantification of central nervous system (CNS) drug levels in brain interstitial fluid (ISF) and cerebrospinal fluid (CSF) is often achieved by complex *in vivo* experimental procedures, such as microdialysis. This technique has the inherent advantage of directly measuring the concentration of unbound drug in the accessible brain biophase under non-steady state and steady-state conditions [1,2], reflecting both drug influx and efflux processes acting within the CNS. To be able to quantify the brain pharmacokinetics of a compound of interest, microdialysis offers the advantage of multiple time-point sampling within the same animal, although the procedure leads to local tissue damage around the site of probe insertion [3,4] and is an experimental procedure often limited to lower-species, although neuroimaging techniques, such as positron emission tomography, have been utilised in both lower- and higher-species to quantify temporal drug concentrations in brain [5]. Microdialysis and PET (positron emission tomography) are often considered the “gold-standard” for assessing (regional) brain disposition of drugs, but can be limiting due to their technical and experimental complexity, which may hinder widespread use in pre-clinical studies.

The ability to determine the relationship between systemic exposure and CNS drug disposition is an important focus for pharmaceutical industry and drug development programs. Typically, pre-clinical measurement of drug partitioning between the CNS (brain tissue and CSF components) and plasma to yield total brain-to-plasma concentration ratio, *K*p_brain_ is conducted in rodents and *K*p_brain_ is then converted to the unbound concentration ratio (*K*p_uu,brain_) by multiplication with plasma unbound drug fraction (*f*u_p_) (Equation (1) *C*, total concentration; *C*_u_, unbound concentration; *V*_u_, unbound brain volume of distribution) [6]. The steady-state unbound brain-to-plasma ratio (*K*p_uu,brain_) (Equation (2)) or steady-state cerebrospinal fluid-to-plasma concentration ratios (CSF_u_:Plasma_u_ and CSF:Plasma_u_) (Equations (3) and (4) respectively) are routinely used to represent CNS disposition of pharmacologically active drugs within the CNS.

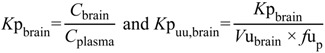
(1)

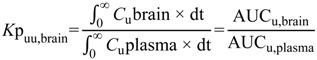
(2)

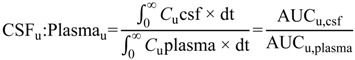
(3)

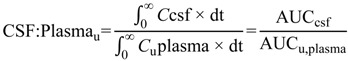
(4)

*K*p_uu,brain_ and CSF_u_:Plasma_u_ values less than 1 typically indicate restricted entry into the brain or CSF-compartments, predominantly a result of efflux or uptake transport proteins respectively, whereas values greater than 1 indicate unrestricted entry into the brain or CSF, facilitated by active transport. Values close to unity indicate predominantly passive transport of drug.

A major factor in successful delivery of drugs to the CNS is circumvention of physiological barriers. The ATP-binding Cassette (ABC) efflux transporters P-glycoprotein [7], breast cancer resistance protein (BCRP) and several multidrug resistance-associated proteins (MRPs) are expressed at the BBB (blood–brain barrier) [7,8,9,10]. *Mdr1* knockout studies in mice reveal that P-glycoprotein significantly influences CNS disposition of both non-CNS targeted and CNS targeted therapeutics including amitriptyline, nortriptyline [11], olanzipine [12], buspirone, chlorpromazine, fluvoxamine, risperidone, zolpidem [13] and fexofenadine [14]. Similar reports of altered brain penetration of imatinib [15], oseltamivir [16] and genistein [17] have been reported in breast cancer resistance protein knockout mice. In addition to BBB-associated ABC transporters influencing CNS drug disposition, expression of highly restrictive tight junction complexes at the BBB (the transcellular electrical resistance is reported to be between 1000 and 1800 Ω cm^2^ [18,19,20]) results in only limited passive diffusion of hydrophilic, low molecular weight (<400 Da) compounds [21] across the BBB into the CNS.

The blood-cerebrospinal fluid barrier (BCSFB) also can regulate entry of compounds into the CNS [22] and is an important consideration when describing CNS drug disposition. The BCSFB is located next to the choroidal epithelium, a continuous single layer of polarized epithelial-like cells, possessing tight junctions [23], which line the surface of the choroid plexuses. There are important physiological differences between the BBB and BCSFB. *In vitro* measurements suggest the transcellular electrical resistance of the BCSFB is approximately 10- to 15-fold less than that of the BBB, at 80–100 Ω cm^2^ [18,19,20]. Unlike the BBB, the choroidal epithelium possesses extensive microvilli and studies suggest the total surface area of the choroid plexuses may be 10-fold greater than previous estimates, placing the surface area within a similar order of magnitude to that of the BBB [24,25,26,27,28], and resulting in *in vivo* BCSFB clearance measurements, per gram of brain, which may be similar to or greater than that at the BBB [29]. However, both P-glycoprotein [30,31] and BCRP [31] have been reported to be expressed at the apical plasma membrane of the choroidal epithelium, and have the potential to transport drugs from the choroidal epithelium into the ventricular CSF. It is therefore important that the differential transport directionalities at the BBB and BCSFB sites are taken into consideration when attempting to predict drug disposition within the CNS.

Efflux transporter proteins at the BBB will therefore limit penetration of compounds into the brain and impact on CNS disposition, whereas efflux transports at the BCSFB will act to potentially enhance the accumulation of compounds in the CSF. Consequently, for highly effluxed drugs there is often a discrepancy between the effects of efflux at the BBB (influencing *K*p_uu,brain_) and the BCSFB (influencing CSF_u_:Plasma_u_) [32,33,34].

Clearly, the measurement of brain unbound concentrations would provide a better indicator for assessing CNS disposition, but microdialysis is not an option routinely employed, pre-clinically. However, determination of the extent of non-specific brain tissue binding (*f*u_brain_), using brain slice and brain homogenate methods, is utilised to drive forward an understanding of overall brain drug penetration. Thus, an understanding of the role of drug transporter proteins at both the BBB and BCSFB coupled with knowledge of brain tissue binding is crucial in order to more effectively predict CNS drug disposition (*K*p_uu,brain_ and CSF_u_:Plasma_u_) and facilitate early pharmacokinetic predictions and selection of compounds for further development [13,35].

A key paradigm in CNS drug development is the prediction of brain accumulation of candidate compounds [36,37]. The application of physiologically-based pharmacokinetic modeling provides an approach to mechanistically incorporate routinely determined *in vitro* data, such as drug permeability and protein binding, into a pharmacokinetic model capable of estimating CNS drug disposition. There is, however, a significant lack of predictive models capable of quantifying CNS drug disposition. In non-physiological models, the CNS is described by either a 1-compartment model (representing brain) or a 2-compartment model (representing brain interstitial fluid and brain intravascular fluid (IVF)) with such models often being used in conjunction with brain microdialysis data to describe CNS drug disposition [2,3]. Semi-physiological models have also been proposed that attempt to mechanistically describe drug disposition within the brain [38,39,40,41,42] but are nonetheless hindered by the requirement for some *a priori* clinically-derived input data.

Recently Ball *et al.* [43] described the development of a whole-body physiologically based pharmacokinetic (PBPK) model for the prediction of unbound drug concentration-time profiles in the rat brain, utilising a mechanistic approach to described drug transfer across the blood–brain barrier. Despite this, there is a lack of fully mechanistic CNS PBPK models employed to describe CNS pharmacokinetics, which limits the application of such models to the prediction of CNS drug disposition.

A key challenge in predicting CNS drug disposition is the extrapolation of cell line-derived permeability data obtained *in vitro* to an *in vivo* permeability metric. *In vitro* permeability data derived from immortalised non-cerebral and cerebral cell lines [44,45,46,47,48,49] has been used previously to assess BBB penetration [50,51,52] despite clear phenotypic differences (e.g., efflux transporter expression profile, enzyme activity) between many of the cell lines used, e.g., Caco-2 (human colorectal adenocarcinoma cell line) and MDCK (Madin-Darby canine kidney cells), and blood–brain barrier endothelial cells.

Recently, positive correlations between drug permeability assessed in the L-mdr1a cell line (the LLC-PK1 porcine kidney cell line transfected with murine P-glycoprotein) and the extent of CNS drug disposition (*K*p_brain_) have been reported [53,54,55]. Of fundamental importance to this correlation is P-glycoprotein protein abundance in transfected cell lines compared to brain microvascular endothelial cells within the BBB. Recent progress in the quantification of absolute expression levels of P-glycoprotein in brain capillaries has estimated total *mdr1a* protein abundance in mouse brain capillaries to be 14.1 fmol/μg protein [56] and rat brain capillaries to be 19.1 fmol/μg protein [57] which is very similar to the *in vitro* protein abundance in L-mdr1a cells, 15.2 fmol/μg protein [54,55], but higher in comparison to that measured in human brain capillaries (6.06 fmol/μg protein) [54]. Such findings suggest data derived from L-mdr1a cells could be incorporated into predictive physiologically-based pharmacokinetic models and may prove useful in assessing CNS drug disposition for P-glycoprotein substrates.

In the present study we describe a predictive, physiologically-based pharmacokinetic model of the rat CNS which incorporates discrete brain and CSF components and is able to predict brain-to-plasma and CSF-to-plasma ratios using *in vitro* permeability parameters and drug protein/tissue binding data. In addition, we also developed a mouse whole-body PBPK model which, when populated with mouse physiological parameters and L-mdr1a cell-derived data, allowed prediction of mouse *K*p_uu,brain_ and CSF:Plasma_u_ (see Supplementary Information).

## 2. Experimental Section

### 2.1. Development of a Whole-Body Physiologically Based Pharmacokinetic (PBPK) Model

#### 2.1.1. Model Development

**Figure 1:**
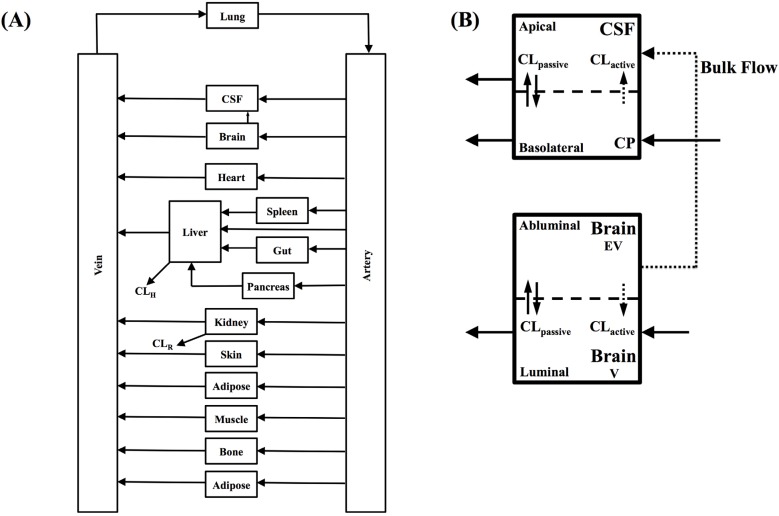
(**A**) Whole-body physiologically based pharmacokinetic (PBPK) model. CL: Clearance; CSF: Cerebrospinal fluid; and (**B**) Brain and CSF compartments. V: vascular compartment; EV: extra-vascular compartment; CL_passive_: passive clearance; CL_active_: active efflux clearance.

A whole-body PBPK model was constructed in Matlab (version 8.1). The model consisted of the following compartments: lung, bone, brain vascular space (V), brain extravascular space (EV), cerebrospinal fluid (CSF), choroid plexus (CP), heart, kidney, liver, muscle, adipose, skin, pancreas, gut, spleen, and arterial and venous blood (Figure 1). All tissue compartments were considered well stirred (perfusion limited) except for CNS-related compartments (Figure 1). 

Mouse and rat tissue volumes and perfusion rates were sourced from literature sources [58] (Table 1) with drug tissue partition coefficients calculated from the tissue-composition-based approach [59,60] using Log*P* and pKa parameters predicted using ChemAxon (http://www.chemaxon.com) or obtained from the literature (see Supplementary Information). Where absent in the literature, blood flow was scaled based on an allometric function (weight^3/4^), and tissue volumes scaled to body weight [61,62], assuming a mouse body weight of 30 g and rat body weight of 250 g [58] (see Supplementary Information).

**Table 1:** Physiologically based pharmacokinetic (PBPK) model parameters for rats and mice.

Tissue	Rat ^a^		Mice ^b^	
	Blood flow	Tissue volume	Blood flow	Tissue volume
	(mL/h)	(mL)	(mL/h)	(mL)
Arterial blood	2580	5.6	839.0	0.40
Venous blood	2580	11.3	839.0	0.81
Lung	2580	1.6	839.0	0.12
Liver (Total)	120	10.3	16.8	0.74
Hepatic artery ^c^	0.29	-	0.28	-
Portal vein ^d^	2.12	-	1.96	-
Kidney	553	2.3	91.9	0.17
Stomach	8	1.1	4.8	0.08
Spleen	37.8	0.6	9.5	0.04
Pancreas	30	1.3	2.2	0.09
Intestine	451	11	117.0	0.79
Muscle	450	122	133.0	8.79
Adipose	24	10	59.0	0.72
Skin	350	40	48.4	2.88
Bone	75.9	15.8	92.3	1.14
Heart	236	0.8	55.1	0.06
Thymus	18	0.7	1.4	0.05
Brain ^e^	120 ^f^	1.8	25.9 ^g^	0.36
Brain IVS ^h^	-	0.025	-	0.005
Brain ISF	-	0.33	-	0.067
ISF bulk flow	0.03 ^i^	-	0.0016	-
CP ^j^	-	0.0036	-	0.00072
CSF	80	1.2	25.8	0.09

^a^ Taken from Brown *et al.* [58]; ^b^ taken from Brown *et al.* [58] or blood flow scaled to the 0.75 of body weight and tissue volumes scaled to body weight (bold) [61,62]; ^c^ assuming hepatic artery flow is 2% (mouse) and 2.1% (rat) of cardiac output [58]; ^d^ assuming portal vein flow is 14.1% (mouse) and 15.3% (rat) of cardiac output [58]; ^e^ fractional volume of brain intravascular fluid, 0.014; Fractional volume of brain interstitial space, 0.188 [63], assuming brain weight of 1.8 g in rats and 0.36 g in mice [64]; ^f^ average of values reported from Eyal *et al.* [65] and Stange *et al.* [66]; ^g^ taken from Jay *et al.* [67]; ^h^ Brain IVS: brain intravascular space; ^i^ taken from Abbott *et al.* [68]; and ^j^ assuming choroid plexus (CP) weight is 0.2% of brain weight [69].

The CNS was comprised of brain IVS (intravascular space), brain ISF and CSF compartments. A rate-limited permeability barrier between the IVS and ISF and IVS and CSF represented the BBB and BCSFB respectively, and was incorporated into the model as passive bi-directional clearance terms (CL_passive_) and active efflux terms (CL_active_) modeling both passive and active flux of compounds across each permeability barrier (Figure 1). Bulk flow of ISF was incorporated within the model to represent the flow of unbound brain ISF drug to CSF. Unbound drug fractions in plasma (*f*u_p_), brain ISF (*f*u_b_) and cerebrospinal fluid (*f*u_CSF_) were incorporated into the plasma, brain and CSF compartments respectively.

Well-stirred organs were described by the following equation:

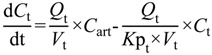
(5)
where *C* is the concentration of drug, *Q*_t_ is the tissue perfusion rate, *C*_art_ is the arterial drug input, *V*_t_ is the volume of tissue compartment and *K*p_t_ is the partition coefficient of the tissue compartment.

The removal of drugs from the eliminating organs (liver and kidney) was described by additional clearance terms (hepatic clearance: CL_H_ and renal clearance: CL_R_). Hepatic clearance was predicted from *in vivo* data (human blood or plasma clearance: CL_b_ or CL_p)_ or *in vitro* data (*in vitro* intrinsic metabolic clearance: CL_int, *in vitro*_) and renal clearance was calculated using a GFR (glomerular filtration rate) correction approach [70].

When using CL_b_ or CL_p_ as an input, the *in vivo* intrinsic clearance (CL_int, *in vivo*_) was calculated (Equation (6)) by, if necessary, correcting for the blood:plasma ratio (*R*_b_) (Equation (7)) (or, where not available, by assuming *R*_b_ = 1 for basic drugs and *R*_b_ = 0.55 for neutral and acidic drugs), and scaled using an allometric function of body weight (weight^3/4^) to yield a species-specific CL_int, *in vivo*_.

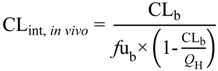
(6)

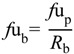
(7)

When using the *in vitro* intrinsic metabolic clearance (CL_int, *in vitro*_) as input, an *in vivo* intrinsic clearance (CL_int, *in vivo*_) term was calculated by scaling CL_int, *in vitro*_ accounting for microsomal recovery (microsomal protein content (rat: 45 milligrams protein per gram of liver [71] or hepatocellularity 130 × 10^6^ cells per gram of liver) [72,73] and rat or mouse liver weight (40 grams per kilogram body weight [58] and 88 grams per kilogram body weight [71] respectively.

The unbound hepatic plasma clearance was then calculated using a well-stirred liver model (Equation (8)), where hepatic blood flow (*Q*_h_) was assumed to be 55 mL min^−1^ kg^−1^ (rats) and 90 mL min^−1^ kg^−1^ (mice) [71].

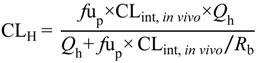
(8)

For compounds which are cleared renally, unbound renal clearance (CL_R_) was predicted using the GFR approach described by Lin [71] and by assuming a rat/human GFR ratio of 4.8 and a mouse/human GFR ratio of 6.6 (Mouse GFR = 12 mL/min/kg [74]), corrected for rat *f*u_plasma_.

Permeability rate-limited transport across the BBB was described by Equations (9) and (10).

Vascular compartment:

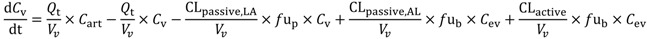
(9)

Extra-vascular compartment:

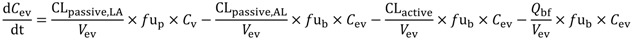
(10)
where *Q*_t_ is tissue perfusion rate, *V*_ev_ is extra-vascular volume, *V*_v_ is the vascular volume, CL_passive_ is the passive clearance across the BBB (subscript denotes either luminal-to-abluminal or abluminal-to-luminal flux), *f*u_p_ is free drug fraction in plasma and *f*u_b_ is free drug fraction in brain. 

Permeability rate-limited transport across the BCSFB was described by Equations (11) and (12).

CP compartment:

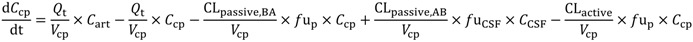
(11)

CSF compartment:

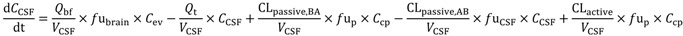
(12)
where *Q*_t_ is the perfusion rate, *V*_cp_ is choroid plexus cellular volume, *V*_CSF_ is the CSF volume, CL_passive_ is the passive clearance across the BCSFB (subscript denotes either basolaterial-to-apical (BA) or apical-to-basolaterial (AB) flux), *f*u_b_ is free drug fraction in brain, *f*u_p_ is free drug fraction in plasma, *f*u_CSF_ is free drug fraction in CSF and *C*_ev_ is the concentration in the brain extravascular compartment.

#### 2.1.2. Extrapolation of Passive Transport

Where apparent permeability (*P*_app_) was reported in the absence and presence of transporter inhibitor, passive transport was assumed to be represented by the extent of inhibition. Where apparent permeability was reported in wild type and knock-out animals, passive transport was assumed to be the difference in apparent permeability. Passive bi-directional transport across the brain capillary was assumed to be represented by the apical-to-basolateral flux (*P*_app,AB_) and basolateral-to-apical flux (*P*_app,BA_) in the non-transfected LLC-PK1 cell line (by correcting for the insert surface area (0.33 cm^2^) and expressed as cm/h), and extrapolated to *in vivo* CL_passive_ for the luminal-to-abluminal (blood-to-brain) and abluminal-to-luminal (brain-to-blood) directions. Passive transport was effectively extrapolated to an *in vivo* passive clearance term based on correction for *in vivo* brain vascular endothelial surface area (SA), 150 cm^2^ g brain^−1^ for rats [75] and 240 cm^2^ g brain^−1^ for mice [44] and brain weight (rat: 0.57% of body weight; mouse: 1.6% of body weight [58]) yielding CL_passive,LA_ (Equation (13)) or CL_passive,AL_ (Equation (14)).

CL_passive,LA_ = *P*app_AB_ × SA × Brain weight (13)

CL_passive,__AL_ = *P*app_BA_ × SA × Brain weight (14)

No studies have directly correlated drug permeability, *in vitro* or *in vivo*, at the BCSFB and the BBB. However, the *in vivo* permeability-surface area product (PS) of quinolone antibiotics at the choroid plexus [76,77] has been modeled in rats, and whilst based on pharmacokinetic modeling approaches, yielded similar *in vivo* permeabilities at the BBB (PS_BBB_) and BCSFB (PS_CSF_), when corrected for tissue weight. Furthermore the paracellular permeability of sucrose in monolayers of primary rat brain endothelial cells (average of 5 studies: 2–11 × 10^−6^ cm/s [78,79,80,81,82], is similar to that reported in monolayers of primary rat choroid plexus cells (7 × 10^−6^ cm/s [83]).

Due to the absence of either *in vitro* or *in vivo* choroidal epithelial permeability data for many compounds, passive flux across the BCSFB was extrapolated based on correcting for *in vivo* choroid plexus surface area (75 cm^2^ in rats [27]) to yield an *in vivo* permeability clearance at the BCSFB (CL_passive, BCSFB_) (Equation (15)):

CL_passive_= *P*_app_ × SA (15)

Bi-directional flux (CL_passive, apical-to-basolaterial_ and CL_passive, basolaterial-to-apical_) and active efflux at the BCSFB was parameterised using a similar approach to that detailed for the BBB.

When using *in vivo* reported CL_passive_ to describe passive permeability at the BBB, CL_passive_ at the BCSFB was scaled based on the BCSFB:BBB surface area.

#### 2.1.3. Extrapolation of Active Transport

Effective extrapolation of *in vitro* determined active transport data requires knowledge of cellular transporter expression within the *in vitro* system and within the target tissue to account for variations in transporter expression. To address this, Ball *et al.* [43] reported an approach that utilised either a relative activity factor (RAF) or a physiological scaling factor to relate activity/expression of transporters within *in vitro* systems to an *in vivo* metric. Furthermore, Hoffmeyer *et al.* [84] suggested that the transport activity of P-glycoprotein in human is dependent on the level of protein expression. Similarly, Shirasaka *et al.* [85] and Tachibana *et al.* [86] also demonstrated that P-glycoprotein transport activity *in vivo* was proportional to its protein expression levels *in vitro*. Given these findings we have assumed *mdr1a* activity is directly related to *mdr1a* protein expression level and the *in vitro* intrinsic transport activity of *mdr1a* (transport rate per *mdr1a* protein) is identical to that *in vivo* in rats. The availability of P-glycoprotein and BCRP efflux kinetics terms is limited for a vast number of compounds in the literature and hinders widespread utilisation of PBPK modeling to assess the brain distribution of drugs. *In lieu* of widespread and robust Michaelis–Menten kinetics parameters for transporter substrates, the active efflux component of drug transport was described by a corrected efflux ratio (ER) [55,87] (Equation (16)) derived from the ratio of the efflux ratio in *mdr1- or BCRP*-transfected cells and vector-transfected control cells.

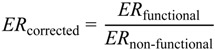
(16)

To correct for the difference in protein abundance between *in vitro* cell lines and brain capillaries, an abundance-scaling factor (ASF) was incorporated to represent the ratio of *in vivo*-to-*in vitro* capillary abundance of transporter protein in cell lines (see Section 2.1.5) and either mice (P-glycoprotein; 14.1 fmol/μg protein or BCRP; 4.41 fmol/μg protein [56]) or rats (P-glycoprotein; 19.1 fmol/μg protein or BCRP; 4.95 fmol/μg protein [57]). For BCRP, *in vitro* abundance data were not available in the MDCK-II-BCRP cell line and therefore ASF was set as equal to 1. Subsequently active clearance was incorporated into the model as the product of the corrected *in vivo* efflux ratio and luminal-to-abluminal passive clearance (Equation (17)).

CL_active_ = *ER*_corrected_ × CL_passive,LA_ × ASF (17)

Active efflux at the BCSFB was modelled using a similar approach, with directionality of efflux transport being from the systemic circulation into the CSF. The proposed model incorporates active efflux for two widely investigated drug efflux transporters, P-glycoprotein and BCRP. Alternative transporter proteins with similar transport directionality could be paramatised within the model using *in vitro* passive and active permeability data for a specific transporter protein along with the protein abundance of the transporter(s).

#### 2.1.4. Model Validation: Prediction of Temporal Brain and Plasma Concentrations in Rats

To validate the PBPK model the plasma and brain concentrations of the antibiotic norfloxacin were modeled and compared to *in vivo* measurements in rats. Norfloxacin plasma pharmacokinetics in rats, following an intravenous (IV) bolus of 150 mg kg^−1^, has been described by a 2-compartment model [88]. For modeling purposes, the unbound fraction of norfloxacin in brain was assumed to be equal to 1. This approach can be rationalised since the unbound brain volume of distribution (*V*_u,brain_) [89] for norfloxacin (0.98 ± 0.59 mL g brain^−1^), is similar to the brain water volume (0.8 mL g brain^−1^) [90] suggesting limited brain binding. Predicted norfloxacin brain ISF- and plasma concentration-time profiles were compared with *in vivo* norfloxacin brain ISF (determined using microdialysis) and plasma concentration-time profiles from 10 rats (pharmacokinetic data provided by Chenel *et al.* [88]).

#### 2.1.5. Prediction of *K*p_uu,brain_ and CSF_u_:Plasma_u_ in Rat

The rat CNS hybrid PBPK model was used to predict *K*p_uu,brain_ and CSF_u_:Plasma_u_. Permeation across the BBB and BCSFB was incorporated into the model using *in vitro* permeability determined in the L-mdr1a cell line, as reported by Uchida *et al.* [55] and detailed in Section 2.1.3. All compounds were simulated as intravenous bolus doses. *K*p_uu,brain_ and CSF_u_:Plasma_u_ were predicted for a dataset of 25 compounds where *in vitro* permeability, *f*u_plasma_, *f*u_brain_, *f*u_CSF_, *K*p_uu,brain_ and CSF_u_:Plasma_u_ had previously been reported in rats [53] (see Supplementary Information).

### 2.2. Prediction of *K*p_uu,brain_ for Actively Effluxed Compounds in Mice

In order to assess the utility of *in vitro*-derived cell culture permeability data to predict CNS drug disposition for actively effluxed compounds in mice, a whole body CNS PBPK model was parameterised with physiological tissue volumes and perfusion rates obtained from literature [91], with any absent data assumed to be equivalent to rats [59,60]. Permeation across the BBB and BCSFB was incorporated into the model using *in vitro* permeability determined in the L-mdr1a cell line, as reported by Uchida *et al.* [55] and the brain disposition of 11 P-glycoprotein substrates was modeled and predictions compared to reported *K*p_uu,brain_ in mice [55]. All compounds were simulated as intravenous bolus doses.

### 2.3. Sensitivity Analysis

To further explore the factors that influence the disposition of drugs into the brain biophase, a series of additional simulations were conducted exploring the impact of variation in CL_passive_ (luminal-to-abluminal and abluminal-to-luminal were assumed equal), *ER*, *f*u_plasma_ and *f*u_brain_ on *K*p_uu,brain_ and CSF_u_:Plasma_u_ utilising input parameters based on a model compound selected from the *K*p_uu,brain_ and CSF_u_:Plasma_u_ predictions.

### 2.4. Assessment of Prediction Accuracy

The predictability of individual compounds was assessed using a fold-error (*FE*) approach where:

Predicted > Observed:

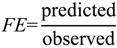
(18)

Observed > Predicted:

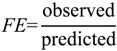
(19)

Prediction accuracy was assessed by the average fold error (*afe*) approach (geometric mean error) (Equation (20)):

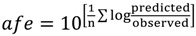
(20)

Precision of prediction was assessed using root mean squared error (*rmse*) (Equation (21)) where *n* refers to the number of observations.

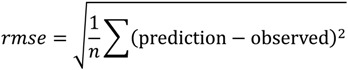
(21)

The percentage of compounds within a 3-fold, 4-fold, 5-fold and >5-fold error was derived from predicted and observed values.

## 3. Results and Discussion

The availability of *in vivo* permeability measurements for candidate compounds undergoing pre-clinical assessment often remains a limiting factor for efficient and effective use of pharmacokinetic models attempting to model CNS drug disposition. Consequently, *in vitro* permeability data for passively and actively transported compounds are often used to extrapolate to *in vivo* permeability. Polli *et al.* [92] demonstrated a linear relationship between brain penetration (Kin) in rat *in situ* brain perfusion studies and apparent permeability in MDCK type-1 cells with a correlation coefficient of 0.86. A similar trend was reported between brain uptake index (BUI) and permeability across bovine brain endothelial cell cultures, with a correlation coefficient of 0.89 [50]. In more recent studies Uchida *et al.* [54] and Kodaira *et al.* [53] have demonstrated the utility of murine-mdr1a-expressing LLC-PK1 cells (L-mdr1a) to reconstruct *K*p_uu,brain_ and CSF_u_:Plasma_u_ for a handful of P-glycoprotein substrates.

Our primary goal was to build upon existing approaches aimed at mechanistically predicting CNS drug disposition and examine the potential application of drug permeability data derived from L-mdr1a cells to predict *K*p_uu,brain_ in mice and both *K*p_uu,brain_ and CSF_u_:Plasma_u_ in rats. Development of a PBPK model capable of predicting CNS drug disposition by extrapolation of *in vitro*-derived data may prove a valuable resource for rapid pre-clinical screening of candidate compounds during development.

### 3.1. Validation of the PBPK Model

To validate the PBPK model structure and the ability to predict both plasma and brain ISF temporal concentrations, we selected norfloxacin as a model compound and utilised published rat norfloxacin plasma data and brain pharmacokinetic data obtained by microdialysis [88].

**Figure 2:**
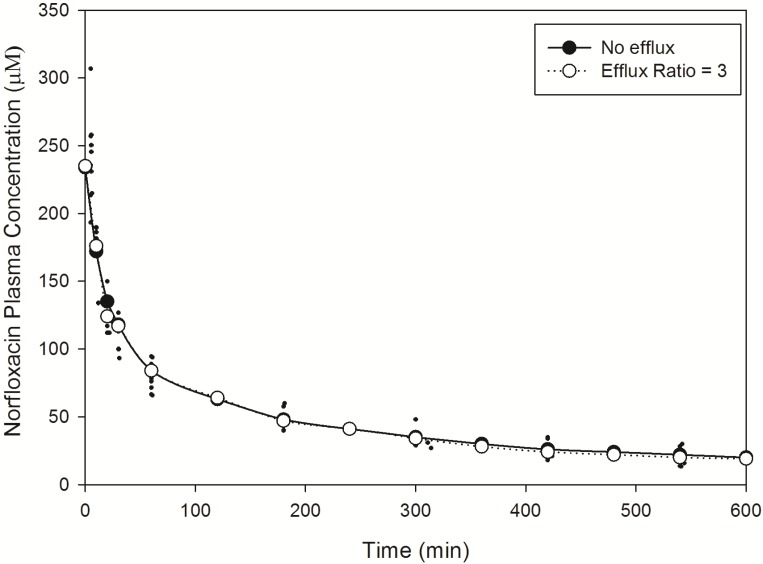
Model predicted norfloxacin plasma concentrations in rats. Small closed circles represent literature reported plasma concentrations determined in rats following an IV-bolus dose [88]. Large closed circles represent model predicted norfloxacin plasma concentrations in rats in the absence of efflux. Large open circles represent model predicted norfloxacin plasma concentrations in rats in the presence of efflux (efflux ratio = 3).

**Figure 3:**
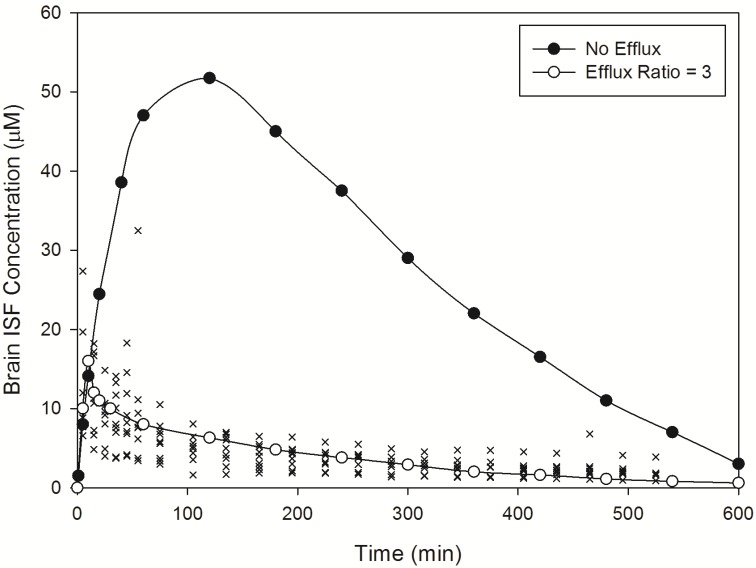
Model predicted norfloxacin brain concentrations in rats. Crosses represent literature reported brain concentration determined in rats following an IV-bolus dose [88]. Closed circles represent model predicted norfloxacin brain concentrations in rats in the absence of efflux. Open circles represent model predicted norfloxacin brain concentrations in rats in the presence of efflux (efflux ratio = 3).

Norfloxacin plasma (Figure 2) and brain (Figure 3) temporal concentration profiles were both predicted to be within the ranges observed *in vivo*. Simulation of brain ISF norfloxacin concentration-time profile using literature derived CL_passive_ (value obtained from fitting to *in vivo* data) [76,77] in the absence of a P-glycoprotein/BCRP-type active efflux component yielded predictions in which the absorption and elimination phases were outside the range observed *in vivo* (Figure 3). Subsequent simulations using a CL_passive_ 2-fold higher than the initial fitted value (Table 2) and P-glycoprotein/BCRP-type active efflux processes (efflux ratio of 3) resulted in absorption and elimination phases within the range reported in 10 rats by Chenel *et al.* [88] (Figure 3).

Importantly, incorporation of an active efflux component (P-glycoprotein/BCRP type) within our simulations corrected the over-prediction in brain ISF drug concentrations and demonstrated the importance of an efflux clearance mechanism in governing norfloxacin CNS drug disposition. These findings are consistent with those of Chenel *et al.* [88] who demonstrated the influence of efflux clearance mechanisms on norfloxacin brain pharmacokinetics. The inclusion of a P-glycoprotein/BCRP type active efflux component within our norfloxacin simulations is supported by a recent report demonstrating norfloxacin to be a BCRP substrate [93].

Norfloxacin *K*p_brain_ was predicted to be 0.141, within 2-fold of the observed *K*p_brain_ of 0.091, whilst norfloxacin CSF:plasma was predicted to be 0.089, within 2.1-fold of the observed CSF:plasma of 0.043 (Table 2). Predicted plasma half-life was extremely close to observed half-life whilst brain ISF half-life was within 1.5-fold of the observed value (Table 2).

**Table 2:** Prediction of norfloxacin plasma, brain and CSF pharmacokinetics.

Parameter	Value	Unit
Predicted *K*p_brain_	0.141	
Mean observed *K*p_brain_	0.093 ^a^	
Predicted CSF:Plasma	0.089	
Observed CSF:Plasma	0.043 ^b^	
Predicted *t*_1/2,plasma_ (*ER* = 3)	183	(min^−1^)
Observed *t*_1/2,plasma_^c^	202 ± 45	(min^−1^)
Predicted *t*_1/2,ISF_ (*ER* = 3)	231	(min^−1^)
Observed *t*_1/2,ISF_^d^	255 ± 97	(min^−1^)
Predicted *AUC*_plasma_	340	(µM min^−1^)
Predicted *AUC*_ISF_ (ER = 0)	329	(µM min^−1^)
Predicted *AUC*_ISF_ (ER = 3)	47.9	(µM min^−1^)
Predicted *AUC*_CSF_ (ER = 3)	30.4	(µM min^−1^)
Predicted ISF *C*_max_ (ER = 0)	52.4	(µM)
Predicted ISF *C*_max_ (ER = 3)	16.3	(µM)

^a^ Mean of three reported values (*K*p_u,brain_: 0.035 ± 0.014 and *K*p_brain_: 0.044 [77], *K*p_brain_: 0.097 ± 0.029 [76] and 0.067 [89]); ^b^ Mean of two reported values (CSF:P_u_, 0.033 ± 0.006 and CSF:P, 0.042 [77], CSF:P_u_, 0.044 ± 0.010 and CSF:P, 0.056 [94]); ^c^ Reported parameter estimate from compartmental analysis [88]; and ^d^ Reported parameter estimate from non-compartmental analysis [95].

### 3.2. Prediction of Central Nervous System (CNS) Disposition Using L-mdr1a in Vitro Permeability Data

Recent studies report positive correlations between drug permeability assessed in the LLC-PK1 porcine kidney cell line transfected with murine *mdr1* (to produce the L-mdr1a cell line) and *in vivo* brain distribution of P-glycoprotein substrates in rats and mice [53].

Furthermore, due to the similarity in the abundance of P-glycoprotein in L-mdr1a cells (15.2 fmol/μg protein) compared to the abundance in brain capillaries (Mouse: 14.1 fmol/μg protein [56]; rat: 19.1 fmol/μg protein [57]), we examined the use of L-mdr1a-derived *in vitro* permeability data in predicting CNS drug disposition.

In an attempt to examine the validity of the scaling approach to determine permeability clearance at the BBB, based on extrapolating *in vitro* permeability data, we obtained literature reported *in situ* brain permeability-surface area products (PS) for 16 compounds spanning over a 100-fold range of PS.

With the exception of three compounds (midazolam, diazepam and sertraline), 11 of 13 compounds fell within 3-fold and 2 within 4-fold of the reported PS values (see Supplementary Information Section 4). Similar trends have been previously reported by Uchida *et al.* (2011) in LLC-PK1 cells [55], Polli *et al.* (2000) [92] and Summerfield *et al.* (2007) [96] in cultured kidney epithelial cells, and support the extrapolation approach.

#### 3.2.1. Prediction of *K*p_uu,brain_ for 11 Actively Transported Compounds in Mice

**Figure 4:**
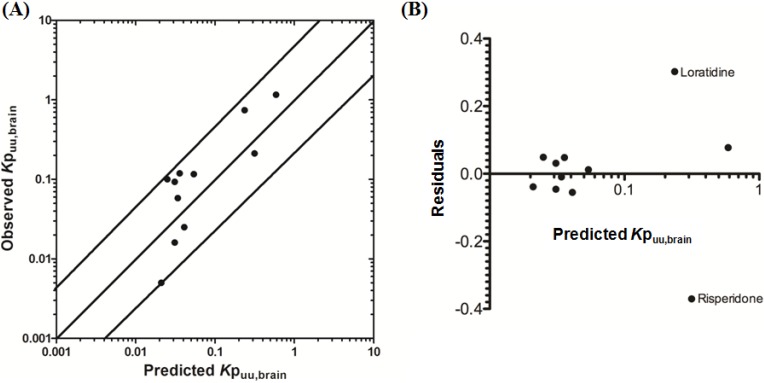
Comparison of predicted and reported *K*p_uu,brain_ in mice. (**A**) Solid bold mid-line represents the line of unity and solid outer-lines represent 4-fold prediction error; and (**B**) residuals plot.

Using L-mdr1a-derived permeability data reported by Uchida *et al.* [55], the predicted *K*p_uu,brain_ for over 90% of P-glycoprotein substrates was within 4-fold of observed *K*p_uu,brain_. The predicted *K*p_uu,brain_ for all compounds was within 5-fold of observed *K*p_uu,brain_ (Figure 4A,B), with an overall *afe* and *rmse* of 0.7 and 0.23 respectively (Table 3).

**Table 3:** Statistics for the model predictions.

Species	Tissue	*n*	*afe*	*rmse*	% within 3-fold	% within 4-fold	% within 5-fold	% > 5-fold
Mouse	Brain	11	0.7	0.23	63.6	90.9	100	0
Rat	Brain	27	1.19	0.43	63	81.5	88.9	11.1
	CSF	27	0.8	0.32	77.8	81.5	96.3	3.7

Uchida *et al.* [55] successfully demonstrated that *K*p_brain_ (and *K*p_uu,brain_) could effectively be reconstructed though the integration of *in vitro* mdr1a transport activity and mdr1a protein expression levels in the brain capillaries and in mdr1a-transfected cell monolayers. Our model yielded reasonable predictions for passively transported and actively transported P-glycoprotein substrates and demonstrated the successful extrapolation of *in vitro* permeability data to yield an *in vivo* transfer clearance across the brain capillaries.

The basis of these predictions is quantitative calculation of the temporal drug concentrations in plasma and brain compartments. Whilst Uchida *et al.* [55] initially reconstructed *K*p_uu,brain_, for the first time we have shown that, using a well-designed PBPK modeling approach, plasma and brain ISF temporal concentrations, and *K*p_uu,brain_ can be adequately predicted in mice for a range of P-glycoprotein substrates, using a simple set of physiochemical and pre-clinically determined parameters.

#### 3.2.2. Prediction of Rat *K*p_uu,brain_ and CSF_u_:Plasma_u_

**Figure 5:**
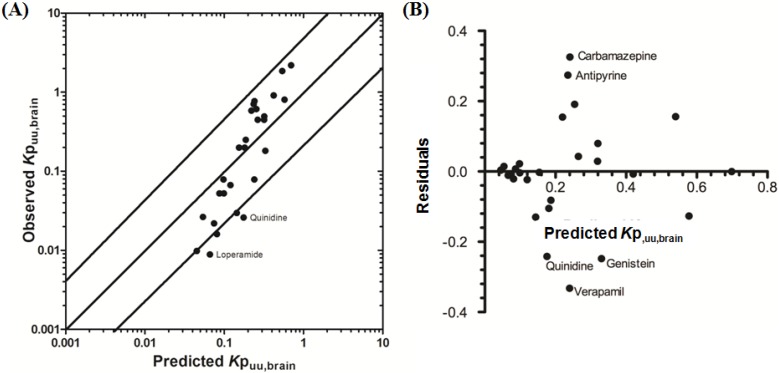
Comparison of predicted and reported *K*p_uu,brain_ in rat. (**A**) Solid bold mid-line represents the line of unity and solid outer-lines represent 4-fold prediction error; and (**B**) residuals plot.

In an attempt to assess the utility of L-mdr1a-derived permeability data to predict cross-species CNS distribution, we utilised L-mdr1a permeability data from 25 compounds to predict *in vivo* CNS distribution (*K*p_uu,brain_ and CSF_u_:Plasma_u_) in rat. Our reported model was capable of predicting rat brain disposition (*K*p_uu,brain_) for 81.5% of compounds simulated to within 4-fold of the reported *K*p_uu,brain_ (Table 3 and Figure 5). The predicted *K*p_uu,brain_ of quinidine was within 6.8-fold of observed *K*p_uu,brain_, whilst that of loperamide within 7.4-fold. The overall *afe* and *rmse* were 1.19 and 0.43 respectively (Table 3).

Predicted *K*p_uu,brain_, for compounds with observed *K*p_uu,brain_ less than 0.01 and greater than 1 deviated further from the line of unity (Figure 5A and local regression (LOESS) plot in Supplementary Information Section 5) but were nevertheless predicted within 4-fold of the reported *K*p_uu,brain_.

For flavopirodol and perfloxacin, the use of either MDCKII or LLC-PK1-derived cell permeability data did not significantly alter model predictions. 

*K*p_brain_ for P-glycoprotein substrates ranges from 1 to 50 [97]. The *K*p_uu,brain_ of quindine and loperamide, typical P-glycoprotein substrates, were 7.4-fold over-predicted in our model. Recent reports have identified a 39.4-fold [55] to 44-fold [53] increase in *K*p_brain_ when comparing wild-type to knock-out mice for quinidine and 23.3-fold [55] for loperamide. For these highly effluxed compounds, the use of *in vitro* permeability data may not truly reflect the extent of *in vivo* efflux and therefore the use of knock-out-to-wild-type *K*p_brain_ (or *K*p_uu,brain_) could also be used as a surrogate metric for efflux. Such an approach improved model predictions of both loperamide (*K*p_uu,brain_ = 0.025) and quinidine (*K*p_uu,brain_ = 0.071) to within a 3-fold prediction window (see Supplementary Information Section 6).

**Figure 6:**
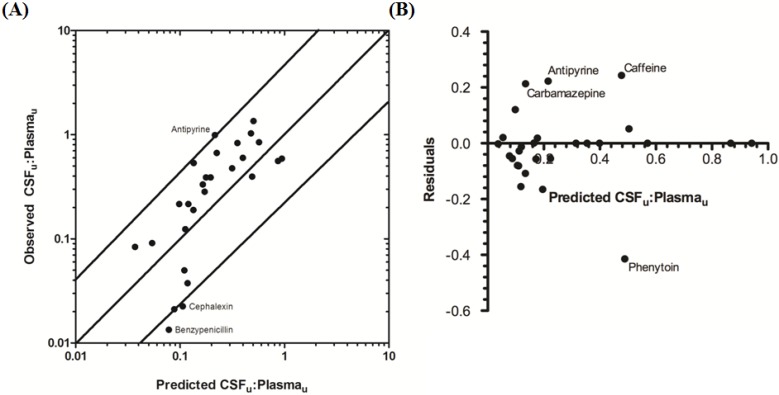
Comparison of predicted and reported CSF_u_:Plasma_u_ in rat. (**A**) Solid bold mid-line represents the line of unity and solid outer-lines represent 4-fold prediction error; and (**B**) residuals plot.

The rat CNS whole-body PBPK model was successful in predicting CSF_u_:Plasma_u_ for 81.5% of compounds to within 4-fold of observed CSF_u_:Plasma_u_ (Table 3 and Figure 6A,B), with CSF_u_:Plasma_u_ of benzylpenicillin 5.8-fold over predicted. The overall *afe* and *rmse* were 0.8 and 0.32 respectively (Table 3).

### 3.3. Model Sensitivity Analysis

Several parameters, particularly passive clearance, active efflux, *f*u_brain_ and *f*u_plasma_, have the potential to significantly impact CNS drug distribution by influencing drug clearance across the BBB and BCSFB. To further explore the relationship between drug clearance across the BBB and BCSFB and the extent of protein/tissue binding, risperidone was selected as a model candidate compound and the impact of variation in passive clearance, active efflux, *f*u_brain_ and *f*u_plasma_ on *K*p_uu,brain_ and CSF_u_:Plasma_u_ was assessed.

#### 3.3.1. Passive Clearance

##### 3.3.1.1. Impact of Variation in *f*u_plasma_ and *f*u_brain_ on *K*p_uu,brain_ and CSF_u_:Plasma_u_

**Figure 7:**
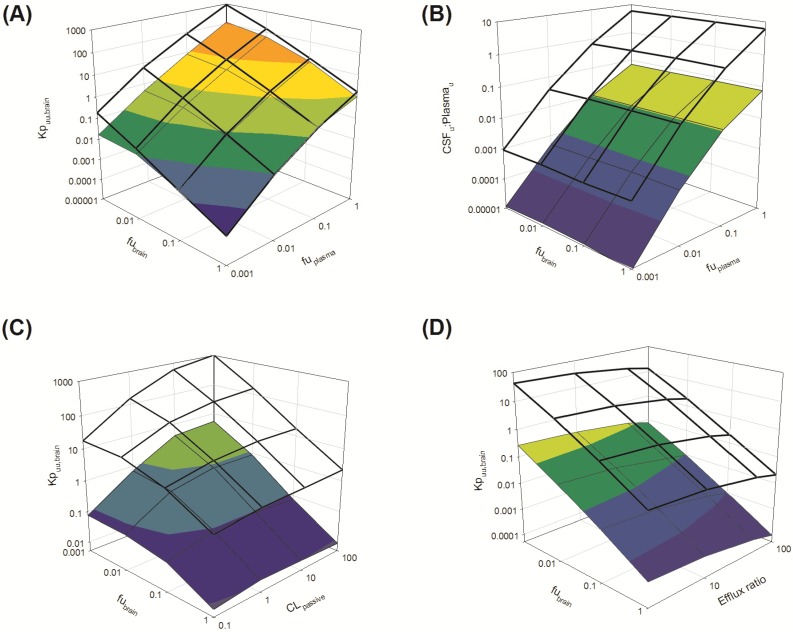
Sensitively analysis of the whole-body physiologically based pharmacokinetic (PBPK) model. The impact of variations in *f*u_brain_, *f*u_plasma_, CL_passive_ and efflux ratio on *K*p_uu,brain_ and CSF_u_:Plasma_u_.

Irrespective of whether the passive clearance (CL_passive_) (*i.e.*, passive permeability) of risperidone at the BBB and BCSFB was low (CL_passive_ 0.34 mL/h) or high (64 mL/h), increasing *f*u_plasma_ (from 0.001 to 1) resulted in a substantial increase in *K*p_uu,brain_ across the range of *f*u_brain_ (0.001 to 1) simulated (Figure 7A: transparent mesh indicates high permeability condition; coloured profile indicates low permeability condition).

Under conditions of both low and high CL_passive_, an increase in *f*u_brain_ (from 0.001 to 1) was associated with a decrease in brain partitioning (*K*p_uu,brain_) of risperidone. This decrease was observed across the range of *f*u_plasma_ (0.001 to 1) simulated (Figure 7A: transparent mesh indicates high permeability condition; coloured profile indicates low permeability condition).

Overall, *K*p_uu,brain_ at high CL_passive_ was greater than *K*p_uu,brain_ at low CL_passive_ when *f*u_brain_ < 0.1.

Brain penetration is therefore influenced by the extent of plasma protein binding (*f*u_plasma_) and the extent of drug binding within the brain (*f*u_brain_). Whilst these observations are relatively intuitive, the importance of both *f*u_plasma_ (and hence unbound drug concentration in plasma) and drug permeability across CNS barriers in influencing CNS drug disposition is clearly demonstrated for drugs that exhibit high non-specific binding to brain tissue (*f*u_brain_). For drugs that are highly bound to brain, *f*u_plasma_ drives entry of drug into the brain. Such drugs are retained within the bulk of the brain (bound-unbound cycling) creating a sink effect, and increasing BBB CL_passive_ would enhance this sink effect further increasing *K*p_uu,brain_ [98,99,100].

The disposition of drug into the CSF was demonstrated to be sensitive to *f*u_plasma_, with increased CSF_u_:Plasma_u_ associated with increasing *f*u_plasma_. This finding was apparent for both low and high CL_passive_ conditions (Figure 7B: transparent mesh indicates high permeability; coloured profile indicates low CL_passive_ conditions). However, simulations were insensitive to any change in *f*u_brain_ (0.001–1) (Figure 7B). These simulations demonstrated no apparent relationship between the extent of *f*u_brain_ and CSF_u_:Plasma_u_, suggesting *f*u_brain_ alone does not significantly influence the unbound concentration of drug within the CSF. These findings support the notion that the extent of free drug in plasma is an important factor influencing drug penetration across the BCSFB into the CSF.

##### 3.3.1.2. Impact of Variation in *f*u_brain_ and CL_passive_ on *K*p_uu,brain_

Irrespective of the extent of plasma protein binding (*f*u_plasma_ 0.01 (low) or 1 (high)), *K*p_uu,brain_ was insensitive to changes in CL_passive_ at higher *f*u_brain_ (*f*u_brain_ > 0.1) (Figure 7C: transparent mesh indicates high *f*u_plasma_; coloured profile indicates low high *f*u_plasma_). The sensitivity of *K*p_uu,brain_ to changes in CL_passive_ increased as *f*u_brain_ decreased (<0.1) (Figure 7C).

As already established, *f*u_plasma_ determines the unbound plasma drug concentration available to penetrate the BBB and BCSBF, where higher *f*u_plasma_ results in an increase in the unbound drug concentration available to cross the BBB and BCSFB. Equally, drug binding in brain provides a driving force for retention of drug within the brain mass, which is evident by the increasing *K*p_uu,brain_ as *f*u_brain_ decreases (irrespective of changes in CL_passive_). However the important role *f*u_brain_ plays in determining *K*p_uu,brain_ for highly brain-bound drugs (*f*u_brain_ < 0.1) is particularly evident for lower permeability compounds (CL_passive_ < 1); *K*p_uu,brain_ appeared not to change significantly when *f*u_brain_ was between 0.001 and 0.1. However *K*p_uu,brain_ was reduced when *f*u_brain_ was between 0.1 and 1 (these findings were observed with both high *f*u_plasma_ and low high *f*u_plasma_ conditions).

#### 3.3.2. Active Clearance

##### 3.3.2.1. Impact of Variation in *f*u_brain_ and Active Efflux on *K*p_uu,brain_

Irrespective of the extent of plasma protein binding (*f*u_plasma_: 0.01 (low) or 1 (high)), *K*p_uu,brain_ was influenced by variations in both *f*u_brain_ over the range studied (*f*u_brain_ 0.001–1) and efflux ratio (2–100) (Figure 7D: transparent mesh indicates high *f*u_plasma_; coloured profile indicates low *f*u_plasma_). *K*p_uu,brain_ increased as *f*u_brain_ decreased from 1 to 0.001, with extensive brain accumulation (*K*p_uu,brain_ greater than 1) when *f*u_plasma_ was high (*f*u_plasma_ = 1) (Figure 7D).

The increase in *K*p_uu,brain_ as *f*u_brain_ decreases can be rationalised by considering that *K*p_uu,brain_ is largely driven by a combination of membrane permeability (passive and active) and drug free fraction in plasma and brain. Where permeability is low (<0.5 mL/h) the impact of variation in *f*u_brain_ on *K*p_uu,brain_ is limited (Figure 7C). When passive permeability increases (CL_passive_ > 0.5 mL/h), and with increasing active efflux at the BBB (Figure 7D), the extent of dug passive permeability may augment *K*p_uu,brain_ and counter the impact a reduction in *f*u_brain_ would have on *K*p_uu,brain_.

##### 3.3.2.2. Impact of Variation in CL_passive_ and Efflux Ratio on *K*p_uu,brain_

**Figure 8:**
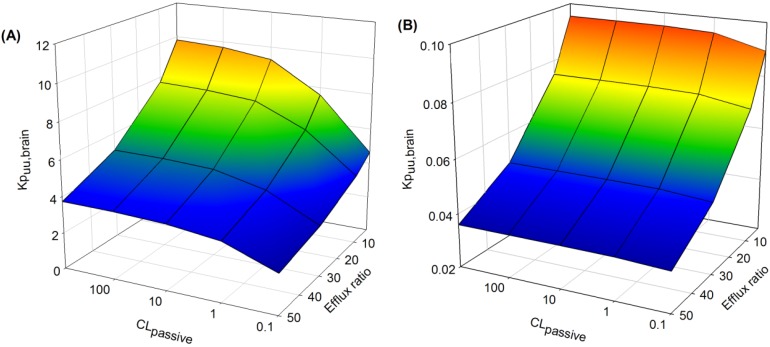
Sensitively analysis of the whole-body PBPK model. The impact of variations in fu_brain_ (**A**) low fu_brain_ and (**B**) high fu_brain_, CL_passive_ and efflux ratio on *K*p_uu,brain_ (see text for details).

The extent of non-specific binding of drug in brain (*f*u_brain_) had a significant effect on the sensitivity of *K*p_uu,brain_ to CL_passive_ and to active efflux (Figure 8). When drug was highly bound in brain (Figure 8A: *f*u_brain_ = 0.01 and *f*u_plasma_ = 1), increasing the extent of drug efflux (efflux ratio 2–50) resulted in a progressive decrease in *K*p_uu,brain_, which was more apparent at higher CL_passive_ (>10 mL/h).

Interestingly, at lower CL_passive_ (<1 mL/h), increasing the extent of active efflux had minimal effects on *K*p_uu,brain_ compared to higher CL_passive_ (>1 mL/h). This effect was diminished when *f*u_brain_ was high (Figure 8B: *f*u_brain_ = 1 and *f*u_plasma_ = 1), since *K*p_uu,brain_ was not sensitive to changes in CL_passive_ over a range of efflux ratios (2–50).

*F*u_brain_ governs the unbound drug concentration in brain and, in conjunction with the clearance of drug across the BBB, helps to regulate the rate and extent of CNS drug accumulation. With extensive non-specific drug binding in brain tissue (Figure 8A), *K*p_uu,brain_ was higher than when *f*u_brain_ is not a limiting factor (Figure 8B). In the absence of an efflux effect the sensitivity of *K*p_uu,brain_ to *f*u_brain_, particularly at low CL_passive_ (Figure 8A), may reflect enhancement of the sink effect as drug is readily able to cross the BBB and accumulate within the brain mass with a diminished abluminal-to-luminal clearance. As active efflux increases, this effect is diminished as efflux provides an additional driving force to rebalance the partition of drug between intravascular spaces and brain biophase.

## 4. Conclusions

With development of therapeutic drugs targeted to the CNS lagging behind development of drugs for other therapeutic areas there is an urgent requirement to better predict CNS drug disposition. The application of brain microdialysis and PET imaging techniques will provide a true quantitative understanding of the temporal (regional) brain concentrations, but the techniques and equipment needed for their applications in understanding CNS drug disposition is often a limiting factor to their widespread use.

To address this issue, we have developed a mechanistic, whole-body physiologically-based pharmacokinetic model incorporating both brain biophase (brain ISF) and cerebrospinal fluid compartments, which provided reasonable estimates of brain-to-plasma and CSF-to-plasma ratios using routinely determined experimental parameters (e.g., *in vitro* permeability, efflux ratio, *f*u_plasma_ or *f*u_blood_ and *f*u_brain_). This model not only allows the simultaneous prediction of brain-to-plasma and CSF-to-plasma ratios and examination of the impact of drug permeability and blood flow on CNS drug disposition, but allows a quantitative prediction of unbound drug concentration within the CNS.

Despite the lack of availability of *in vitro* permeability data from representative *in vitro* choroid plexus cell models (such as the immortalised Z310 rat cell line [101]), the model adequately predicted CSF-to-plasma ratios for over 90% of the compounds simulated. The lack of predictive models currently capable of quantifying both brain biophase and CSF drug disposition significantly hinders the assessment of drug disposition within the CNS. Current methods utilising CSF drug kinetics as surrogates for brain drug kinetics remain controversial [95,102], with many studies disagreeing with the use of CSF as a surrogate for brain [103,104,105]. The physiological differences between the BCSFB and the BBB, advocate the viewpoint that CSF and BCSFB are distinct entities when compared to the BBB. In particular, since CSF drug concentrations do not accurately reflect brain drug concentrations for many actively transported compounds, it is essential that the brain and CSF be considered as separate entities within mechanistic models.

Clearly, in the context of the interactions of drug substrates with transporter proteins, the benefit of the proposed PBPK model would be to effectively incorporate the impact of temporal concentrations on transporter activity and the impact this would have on CNS pharmacokinetics.

The proposed model is capable of predicting temporal CNS drug concentrations, however due to the lack of routinely available transporter-specific Michaelis–Menten terms for drug substrates, the proposed approach of examining overall CNS disposition (*K*p_uu,brain_ and CSF_u_:Plasma_u_) is a valid one. In addition, the complexity of modeling the kinetics of drug-transporter protein interaction, at a cellular level, is now recognised and could potentially be examined further within the proposed model if BBB and BCSFB cellular compartments were expanded towards a semi-systems biology based model [106]. It is prudent to note however, that such approaches would benefit from the use of microdialysis or PET imaging in combination with more elaborate semi-systems biology models, to aid in the development and validation of models.

The present study reports, for the first time, a PBPK CNS model that predicts *K*p_uu,brain_ and CSF:Plasma (bound and unbound) for compounds possessing diverse pharmacokinetic characteristics. Additionally, this study illustrates the potential use of *in vitro* L-mdr1a-derived permeability data to predict rat CNS drug disposition within an acceptable tolerance.

## Figures and Tables

**Figure S1:**
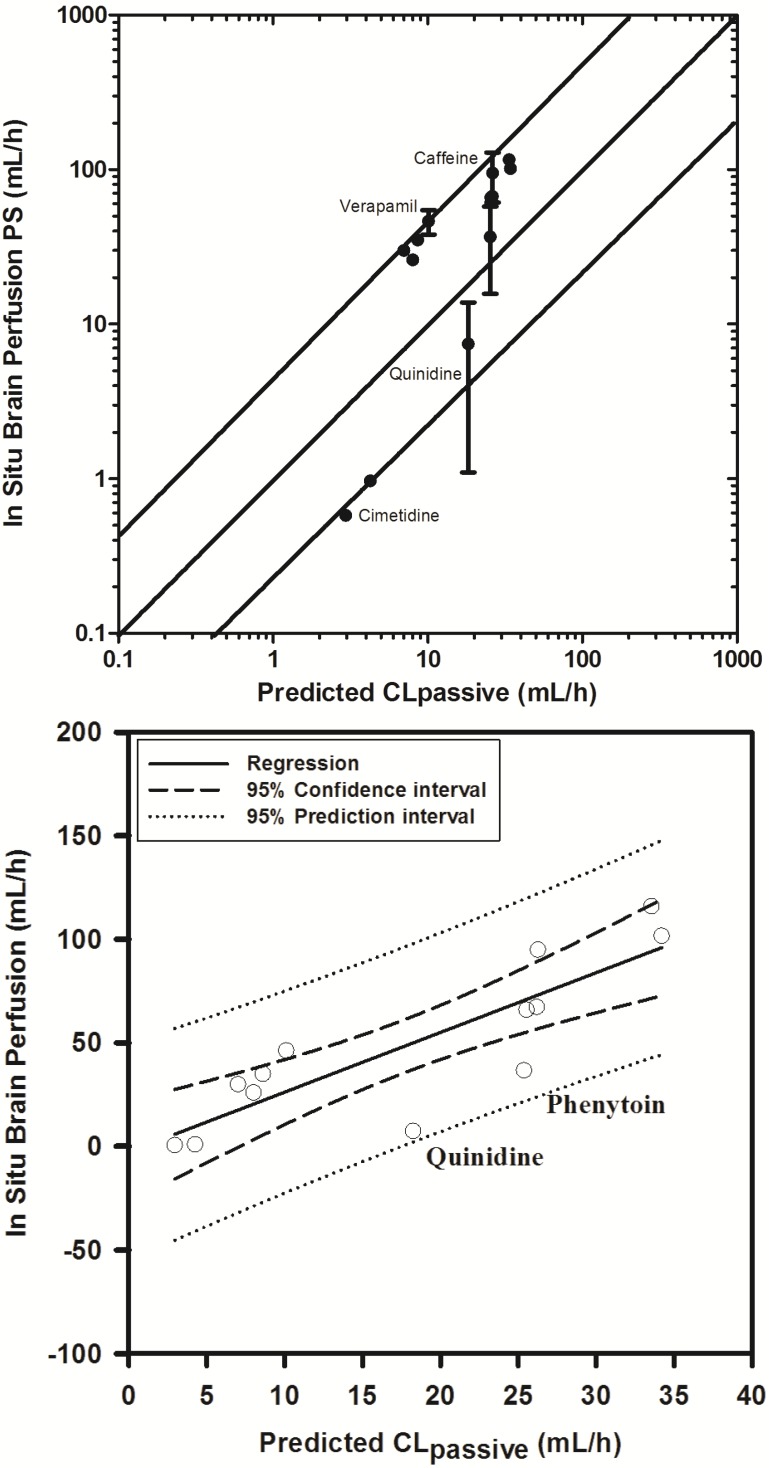
Correlations and confidence interval plots of *in situ* brain perfusion and predictions of CL_passive_.

**Figure S2:**
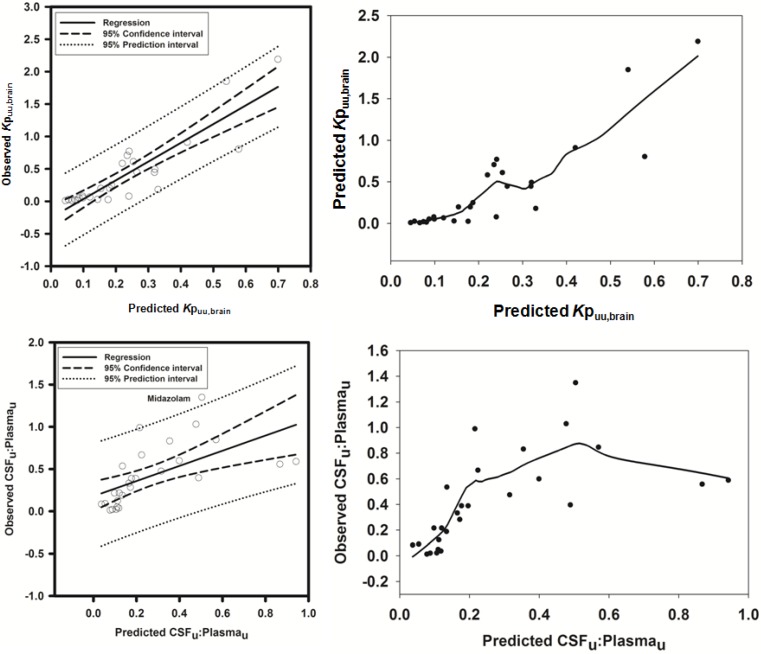
LOESS regression and confidence interval plots.

**Figure S3:**
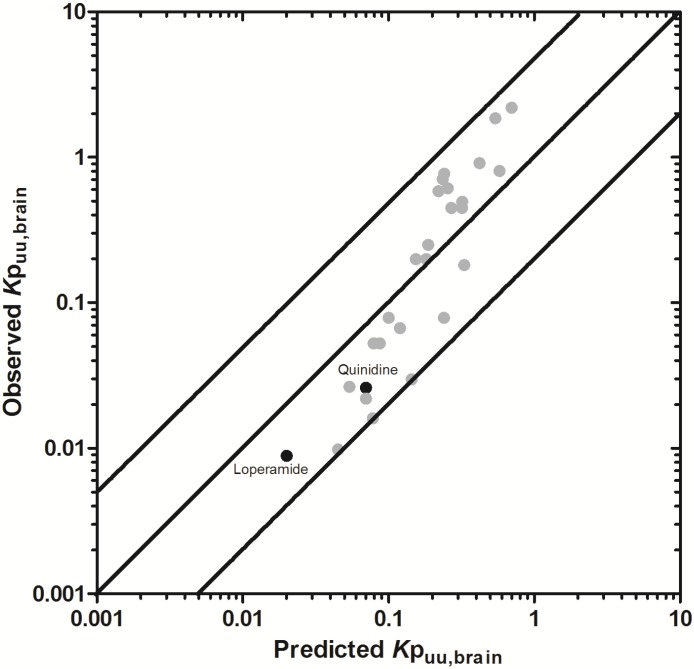
*K*p_uu,brain_ model predictions for loperamide and quinidine (highlighted in black) using an *in vivo* surrogate efflux ratio metric for highly effluxed transporter substrates.

## Supplementary Information

### S1. Model Validation: Prediction of Norfloxacin Temporal Brain and Plasma Concentrations in Rats

**Table S1:** Model input parameters.

Parameter	Value	Units
*Permeability Clearance* ^a^		
CL_passive_	0.21	mL/h
*Efflux* ^b^		
Efflux ratio	3	
*Unbound fraction*		
*f*u_plasma_^c^	0.78	
*f*u_brain_^d^	1	
*f*u_CSF_^e^	1	
*Physicochemcial*		
Log*P* ^f^	−1.03	
pKa ^g^	6.4 and 8.7 (Zwitterion)	
*Total plasma clearance*		
CL ^h^	15.5	mL/min/kg

^a^ Mean of reported values from Ooie *et al.* [76]; ^b^ Reported as apical-to-basolateral/basolateral-to-apical flux [107]; ^c^ Taken from Ooie *et al.* [77]; ^d^ Estimated from *V*_u,brain_ [89]; ^e^ Total CSF concentrations were simulated as only CSF:Plasma_u_ have been reported in literature; ^f^ Taken from Hansch *et al.* [108]; ^g^ Calculated using ChemAxon; and ^h^ Total plasma clearance [88] is split between hepatic (85%) and renal (15%) clearance [109].

### S2. Mouse PBPK Model Compound Specific Parameters

**Table S2:** Mouse PBPK model parameters.

Compounds	*In vitro* permeability ^a^				Active efflux			Permeability clearance		
	P_app_ (L-mdr1a)		P_app_ (LLC-PK1)		Efflux ratio			mL/h		
	10^−6^ cm/s		10^−6^ cm/s							
	A to B	B to A	A to B	B to A	*ER*_functional_	*ER*_non-functional_	*ER*_corrected_	CL_passiveLA_	CL_passiveAL_	CL_active_
Quininde	3.16	146	57.2	80.5	46.2	1.4	30.2	14.8	20.9	447.8
Loperamide	5.49	271	49.7	98	49.4	2	23	12.9	25.4	296.7
Digoxin	1.13	31.9	11.8	18.8	28.2	1.6	16.3	3.1	4.9	49.9
Risperdone	23.3	150	96.3	58.7	6.4	0.6	9.7	25	15.2	242.5
Indindavir	3.33	45.3	14.4	20.5	13.6	1.4	8.8	3.7	5.3	32.8
Dexamethasone	6.91	102	29.5	36.5	14.8	1.2	11	7.6	9.5	83.9
Vinblastine	2.83	56.1	24.2	38.7	19.8	1.6	11.4	6.3	10	71.5
Paclitaxcel	2.7	53.8	20.5	33.2	19.9	1.6	11.3	5.3	8.6	60.1
Verapamiol	11.9	84	73.5	39	7.1	0.5	12.2	19.1	10.1	233.2
Loratidine	32	80.2	23.1	33.1	2.5	1.4	1.6	6	8.6	9.6
Diazepam	57.3	67.2	31.8	43.5	1.2	1.4	0.8	8.2	11.3	6.5

^a^ Taken from Uchida *et al.* [55].

**Table S3:** Mouse PBPK metabolic and renal clearance.

Compounds	Metabolic clearance		Renal clearance	
	Human ^a^	Mouse ^b^	Human	Mouse
	CL_plasma_ or CLint_,u_	CLint_, *in vivo*_	CL_R_	
	mL/min/mg protein or mL/min/kg	mL/min/kg	mL/min/kg	
Quininde	4.02	11.02	0.8	5.28
Loperamide	0.1775	183.56	na	na
Digoxin	0.07	0.08	2.06 ^c^	13.596
Risperdone	5.4	10.76	12.8 ^d^	84.48
Indindavir	14.7	308.30	na	na
Dexamethasone	3.91	6.31	na	na
Vinblastine	15.4 ^e^	221.51	na	na
Paclitaxcel	na	0.54 ^f^	na	na
Verapamiol	13.3	119.58	na	na
Loratidine	na	na	na	na
Diazepam	0.51	1.88	na	na

^a^ unless otherwise indicated, human clearance values were obtained from Hallifax *et al.* [110]; ^b^ unless otherwise indicated, mouse clearance was determined from human clearance (via calculation of human *in vivo* intrinsic hepatic clearance) and allometrically scaled (Human = 70 kg, Mouse = 0.020 kg), see Section 2.1.1 for further details; ^c^ taken from Hedman *et al*. (1990) [111]; ^d^ taken from Thyssen *et al.* [112]; ^e^ taken from Rataom *et al.* [113]; ^f^ taken from Eiseman *et al.* [114]; na: not available.

### S3. Mouse Brain PBPK Model Predictions

**Table S4:** Mouse PBPK model predictions.

Compounds	Predicted	Observed ^a^	Fold error
	*K*p_uu,brain_	*K*p_uu,brain_	
Quinidine	0.034	0.058	1.7
Loperamide	0.031	0.016	1.9
Digoxin	0.021	0.005	4.2
Risperidone	0.315	0.212	1.4
Indinavir	0.036	0.119	3.3
Dexamethasone	0.031	0.093	3
Vinblastine	0.041	0.025	1.6
Paclitaxel	0.054	0.116	2.1
Verapamil	0.025	0.100	4
Loratidine	0.236	0.740	3.1
Diazepam	0.588	1.160	1.9

^a^ Taken from Uchida *et al*. [55].

### S4. Rat CNS PBPK Model

**Table S5:** Permeability data.

Compounds	Transporter	Transporter expressing cells ^a^		Parental cells		Active efflux ^b^		
		P_app_ (μL/h/well)		P_app_ (μL/h/well)		Efflux ratio		
		A to B	B to A	A to B	B to A	*ER*_functional_	*ER*_non-functional_	*ER*_corrected_
Antipyrine		28.7	25.6	31.2	29.6			
Benzypenicillin		7.5	4.7	5.2	3.7			
Buspirone		38.6	38.4	40.7	39.9			
Caffeine		29.7	35.4	32.1	34.5			
Carbamazepine		40.6	45.2	41	46.5			
Cephalexin		4.8	3.7	4	3.7			
Citalopram		25.9	39.2	32	35.5			
Cimetidine	BCRP	1.8	7.2	1.2	2.4	4.0	2.0	1.8
Daidzen	BCRP	16.9	36.4	20.5	24.1	2.2	1.2	1.7
Dantrolene	BCRP	19.9	41.9	31.1	36.1	2.1	1.2	1.7
Diazepam		46.5	42.2	44.4	43.7			
Flavopiridol	P-gp	9.6	67.3	26.6	39.6	7.0	1.5	4.3
Flavopiridol	BCRP	42.9	64	54.9	53.6	1.5	1.0	1.4
Fleroxacin	BCRP	5.6	9.9	5.6	6.8	1.8	1.2	1.3
Genistein	BCRP	13.8	32.4	20.7	23.1	2.3	1.1	1.9
Loperamide	P-gp	12.9	54.7	22.1	15	4.2	0.7	5.7
Midalzolam		38.1	44.5	41.4	42.2			
Pefloxacin	P-gp	5.1	12.3	5.4	8.5	2.4	1.6	1.4
Pefloxacin	BCRP	10.8	17.4	9	9.2	1.6	1.0	1.5
Phenytoin		29.6	37.1	31	37.7			
Quinidine	P-gp	6.6	42.2	22.3	22.9	6.4	1.0	5.7
Risperidone	P-gp	21.3	63	41.8	45.9	3.0	1.1	2.5
Sertraline		2.8	2.4	2.7	1.9			
Sulpiride		2	2.7	1.9	2.4			
Thiopental		39.6	37.7	37.1	36.4			
Verapamil	P-gp	15.4	49	24.9	23.4	3.2	0.9	3.1
Zolpidem		40.3	45.8	42.4	41.6			

^a^ Compounds known to be subjected to active efflux as a result of a transporter protein are indicated by either BCRP (breast cancer resistance protein or P-gp (P-glycoprotein); and ^b^ calculated only for compounds reported to be subjected to active efflux.

**Table S6:** Model predicted *versus* literature reported *in situ* permeabilities.

Compounds	Transporter	Permeability clearance			*In situ* permeability ^b^
			mL/h		mL/h
		CL_passiveLA_^a^	CL_passiveAL_	CL_active_	
Antipyrine		25.5	24.2		66 ± 2.5 [115,116,117]
Benzypenicillin		4.3	3		0.97 [118]
Buspirone		33.3	32.6		
Caffeine		26.3	28.2		95 ± 33.7 [110,115,116,119]
Carbamazepine		10.6	33.5		116 [96]
Cephalexin		3.3	3		
Citalopram		26.2	29		67.3 [96]
Cimetidine	BCRP	2.9	16.5	5.2	0.58 [120]
Daidzen	BCRP	13.5 (7)	32	23	21.6 (WT), 30 (−/−) [121]
Dantrolene	BCRP	18.4 (8.6)	31.6	31.3	16.7 (WT), 35 (−/−) [121]
Diazepam		36.3	35.8		351 ± 254 [96,100,122]
Flavopiridol	P-gp	21.8	32.4	181.1	
Flavopiridol	BCRP	44.9	43.9	62.9	
Fleroxacin	BCRP	4.1	5	5.3	
Genistein	BCRP	14.6	27.1	27.7	
Loperamide	P-gp	18.1 (8)	12.3	130	2 (WT), 26 (−/−) [123]
Midalzolam		33.9	34.5		459 [96]
Pefloxacin	P-gp	4.4	7	7.8	
Pefloxacin	BCRP	7.4	7.5	11.1	
Phenytoin		25.4	30.8		36.7 ± 21 [96,115,124]
Quinidine	P-gp	18.2	18.7	130.7	7.45 ± 6.35 [116,120,125]
Risperidone	P-gp	34.2	37.6	107.7	101.7 [96]
Sertraline		2.2	1.6		129 [96]
Sulpiride		1.6	2		
Thiopental		30.4	29.8		
Verapamil	P-gp	20.4 (10.1)	19.1	79.7	6 ± 0.78 (WT) 46.3 ± 8.42 (−/−) [124,126]
Zolpidem		34.7	34		

^a^ Permeabilities in italics represent *in vivo* passive influx in mice and are used in conjunction with the equivalent *in situ* brain permeability (italics) for correlation purposes; and ^b^ values in italics represent *in situ* brain permeabilities in mice used for correlation purposes.

**Table S7:** Physicochemical parameters used to calculate partition coefficients.

Compounds	Physicochemical parameters ^a^	
	pKa	Log*P*
Antipyrine	1.4	0.38 ^b^
Benzypenicillin	3.55 ^c^	2.74 ^d^
Buspirone	7.62	1.95 ^e^
Caffeine	14	0.92 ^f^
Carbamazepine	15.96	2.1
Cephalexin	3.45 ^g^	0.65
Citalopram	9.78	3.5
Cimetidine	6.8 ^h^	0.26
Daidzen	8.69	0.71
Dantrolene	7.5	1.65
Diazepam	3.4 ^d^	2.82 ^i^
Flavopiridol	5.68	2.8
Fleroxacin	7.15	1.84
Genistein	6.35	3.04
Loperamide	8.6	5.5
Midalzolam	6.2	3.89
Pefloxacin	8.3	0.27 ^c^
Phenytoin	8.3 ^j^	2.5
Quinidine	8.56	3.4 ^c^
Risperidone	8.8	2.5
Sertraline	9.16 ^k^	5.1
Sulpiride	9.12 ^l^	0.57
Thiopental	7.55 ^i^	2.85 ^c^
Verapamil	8.9 ^i^	3.7 ^c^
Zolpidem	6.2	1.2

^a^ Unless otherwise stated, calculated using ChemAxon; ^b^ Stevenson *et al.* [127]; ^c^ Hansch *et al.* [108]; ^d^ Merck Index [128]; ^e^ Ullrich and Rumrich [129]; ^f^ Martin *et al.* [130]; ^g^ Streng W.H. [131]; ^h^ Tomlinson and Hafkenscheid [132]; ^i^ Sangster [133]; ^j^ McLure *et al.* [134]; and ^k^ Deak *et al.* [135]; ^l^ El Tayer *et al.* [136].

**Table S8:** Protein binding and metabolic clearance.

Compounds	Protein Binding ^a^			Metabolic Clearance	
	Plasma	Brain	CSF	Human ^b^	Rat ^c^
	*f*u_plasma_	*f*u_brain_	*f*u_CSF_	CL_plasma_ (mL/min/kg)	CL_int, *in vivo*_ (mL/min/kg)
Antipyrine	0.98	0.86	1	0.57	0.21
Benzypenicillin	0.649	2.26	0.998	na	na
Buspirone	0.45	0.137	0.996	17.17	95.34
Caffeine	0.917	0.697	1	1.67	0.70
Carbamazepine	0.385	0.17	0.995	0.4	0.37
Cephalexin	1.05	1.42	1	na	na
Citalopram	0.82	0.437	0.999	4.5	2.50
Cimetidine	0.529	0.0197	0.997	3.2	2.55
Daidzen	0.0542	0.0828	0.95	na	na
Dantrolene	0.117	0.0746	0.978	na	na
Diazepam	0.211	0.0426	0.989	0.51	0.88
Flavopiridol	0.254	0.0552	0.991	2.1	3.27 ^d^
Fleroxacin	0.793	0.555	0.999	na	na
Genistein	0.0101	0.0531	0.773	na	na
Loperamide	0.0701	0.00196	0.962	0.1775	0.90
Midalzolam	0.045	0.0431	0.94	6.5	75.66
Pefloxacin	0.86	0.514	1	na	na
Phenytoin	0.302	0.0967	0.993	0.47	0.56
Quinidine	0.454	0.0242	0.996	4.02	3.92
Risperidone	0.158	0.0596	0.984	5.4	16.55
Sertraline	0.0347	0.00038	0.923	27.5	158.01 ^e^
Sulpiride	0.88	0.345	1	0.08	0.03 ^f^
Thiopental	0.202	0.244	0.988	na	na
Verapamil	0.101	0.0165	0.974	13.3	138.68
Zolpidem	0.267	0.265	0.992	4.3	7.47

^a^ Taken from Kodaira *et al.* [53]; ^b^ unless otherwise indicated data was taken from Hallifax *et al.* [113], as either plasma clearance (denoted by: p) or microsomal clearance (denoted by: m); ^c^ unless otherwise indicated, intrinsic *in vivo* clearance was calculated based on a well stirrer liver model assuming average hepatic blood flow (*Q*_H_, 55 mL/min/kg). Blood clearance and unbound fraction in blood were determined using the blood:plasma ratio (*R*_b_) or by assuming a value of 1 for basic and neutral drugs and 0.55 for acidic drugs; ^d^ taken from Blum *et al.* [137]; ^e^ calculated from Ronfield *et al.* [138]; and ^f^ taken from Bres *et al.* [139]; na: not applicable.

**Table S9:** Renal clearance. Renal clearance in rats (CL_R_) was calculated based on glomerular filtration rate (GFR) ratio approach as described by Lin [70].

Compounds	Human	Rat
	CL_R_ (mL/min/kg)	CL_R_ (mL/min/kg)
Antipyrine	0.038 ^a^	0.11
Benzypenicillin	3.52 ^b^	10.22
Buspirone	na	na
Caffeine	0.0073 ^c^	0.021
Carbamazepine	na	na
Cephalexin	2.85 ^d^	8.28
Citalopram	na	na
Cimetidine	4.34 ^e^	12.6
Daidzen	na	na
Dantrolene	na	na
Diazepam	na	na
Flavopiridol	na	na
Fleroxacin	0.93 ^f^	2.7
Genistein	na	109 ^g^
Loperamide	na	na
Midalzolam	na	na
Pefloxacin	na	na
Phenytoin	na	na
Quinidine	0.8 ^h^	2.32
Risperidone	na	na
Sertraline	na	na
Sulpiride	1.72 ^i^	4.99
Thiopental	na	na
Verapamil	na	na
Zolpidem	na	na

^a^ Taken from Scavone *et al.* (1989 [140], 1997 [141]) and Thompson *et al.* [142]; ^b^ taken from Rumble *et al.* [143]; ^c^ taken from Birkett and Miners [144]; ^d^ taken from Brogard *et al.* [145]; ^e^ taken from Larson *et al.* [146]; ^f^ taken from Stuck *et al.* [147]; ^g^ taken from Setchell *et al.* [148]; ^h^ taken from Hughes, Ilett and Jellett [149] and Verme *et al.* [150]; and ^i^ taken from Bres and Bressolle [140].

### S5. Rat Brain PBPK Model Predictions

**Table S10:** Model predictions.

Compounds	Transporter	*K*p_uu,brain_^a^			CSF_u_:Plasma_u_^b^		
		Predicted	Observed	Fold error	Predicted	Observed	Fold error
Antipyrine		0.235	0.708	3.0	0.215	0.99	4.6
Benzypenicillin		0.054	0.0264	2.0	0.078	0.0134	5.8
Buspirone		0.254	0.612	2.4	0.867	0.558	1.6
Caffeine		0.220	0.584	2.7	0.477	1.03	2.2
Carbamazepine		0.241	0.771	3.2	0.135	0.535	4.0
Cephalexin		0.078	0.016	4.8	0.106	0.0225	4.7
Citalopram		0.045	0.00981	4.6	0.088	0.0211	4.1
Cimetidine	BCRP	0.320	0.494	1.5	0.224	0.667	3.0
Daidzen	BCRP	0.120	0.0667	1.8	0.134	0.189	1.4
Dantrolene	BCRP	0.144	0.0297	4.8	0.037	0.0838	2.3
Diazepam		0.578	0.805	1.4	0.570	0.847	1.5
Flavopiridol	P-gp	0.087	0.0525	1.7	0.120	0.216	1.8
Flavopiridol	BCRP	0.079	0.0525	1.9	0.098	0.216	2.2
Fleroxacin	BCRP	0.187	0.25	1.3	0.172	0.283	1.6
Genistein	BCRP	0.33	0.181	1.8	0.942	0.589	1.6
Loperamide	P-gp	0.066	0.00886	**7.4**	0.118	0.0376	3.1
Midalzolam		0.699	2.19	3.1	0.504	1.35	2.7
Pefloxacin	P-gp	0.154	0.199	1.3	0.196	0.389	2
Pefloxacin	BCRP	0.181	0.199	1.1	0.177	0.389	2.2
Phenytoin		0.319	0.447	1.4	0.489	0.396	1.2
Quinidine	P-gp	0.176	0.026	**6.8**	0.054	0.0911	1.7
Risperidone	P-gp	0.10	0.0787	1.2	0.112	0.124	1.1
Sertraline		0.54	1.85	3.4	0.354	0.832	2.3
Sulpiride		0.07	0.0219	3.4	0.110	0.0499	2.2
Thiopental		0.42	0.911	2.2	0.399	0.599	1.5
Verapamil	P-gp	0.24	0.0786	3.1	0.165	0.333	2
Zolpidem		0.27	0.447	1.7	0.315	0.475	1.5

^a,b^ Bold indicates parameters predicted with a fold-error >5.
